# Upadacitinib therapy for foreign body granuloma induced by facial fillers^؆^

**DOI:** 10.1016/j.abd.2026.501375

**Published:** 2026-06-16

**Authors:** Mengying Duan, Yi Wu, Hong Shen, Yeqin Dai

**Affiliations:** The Third Affiliated Hospital, Zhejiang Chinese Medical University, Hangzhou Third People’s Hospital, Zhejiang Chinese Medical University, Hangzhou, Zhejiang, China

Dear Editor,

With people's continuous pursuit of facial rejuvenation, a large number of medical cosmetic injection products have emerged. However, the increasing injection complications correspond to a rise in the popularity of facial filler injections. Facial Foreign Body Granuloma (FBG) is one of them, which greatly affects the appearance and psychology of patients. We report a case of a patient who was successfully treated facial FBG caused by dermal fillers with the JAK inhibitor, Upadacitinib.

A 51-year-old woman presented with facial swelling and stiffness. Symptoms gradually developed after she received a facial filler injection (materials unknown) one year ago, followed by extensive facial swelling, mainly involving the forehead, eyelids, and cheeks (Fig. 1A). Oral clarithromycin and intravenous dexamethasone were administered for treatment, but there was minimal improvement. Facial ultrasound (Fig. 1B) revealed a large area of hyperechoic region in the forehead. An enhanced Magnetic Resonance Imaging (MRI) (Fig. 2) of the facial soft tissues showed thickening of the subcutaneous soft tissues in the frontal-temporal region and around the orbits, considered as inflammatory lesions. A skin biopsy (Fig. 3A) revealed the presence of proliferative fibrous tissue in the frontal area, with a large number of lymphocytes, and multinuclear giant cells infiltrated in the dermis, considering a pathological change of FBG. Immunohistochemistry showed CD68 (+), CD163 (a small amount +), lysozyme (+), CD1a (-), S-100 (-), and Ki67 (10%‒15% +). Special staining results were as follows: Alcian blue staining, PAS staining, reticular fiber staining and V-G staining: negative. Combining the medical history, the affected area is consistent with the injection site. By ultrasound, MRI, pathological and immunohistochemical results, the diagnosis was FBG caused by dermal fillers. After coming to the hospital, intramuscular injection of betamethasone was given once every half month; doxycycline hydrochloride, 0.1 g, twice daily; spironolactone tablets, 20 mg, twice daily were administered for treatment. However, the patient didn’t respond to those treatments. Then the patient was given upadacitinib, 15 mg, once daily after no abnormal results were found in routine laboratory tests, including those related to infectious diseases. She returned for a follow-up visit with the resolution of facial swelling within three weeks (Fig. 3B). We did an enhanced MRI scan (Fig. 4) again, which illustrates the regression of the thickening subcutaneous soft tissue. Together with the subsidence of the patient's facial swelling sensation and the MRI results, upadacitinib showed good efficacy. The treatment lasted for 3-months to prevent recurrence. As of now, the patient has not had a recurrence within 5-months.

**Figure 1:**
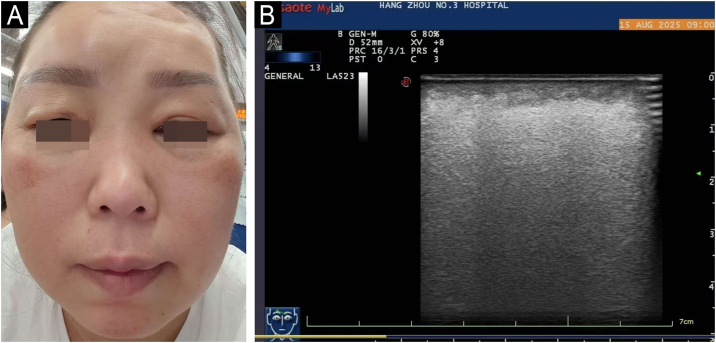
(A) Pretreatment clinical image. (B) Hyperechoic region extended approximately 20 mm above the hairline, involving the root of the nose, the temporal area and the orbits. The deep echo could not be displayed. Doppler Flow Imaging (CDFI) showed no obvious blood flow signal within it.

**Figure 2:**
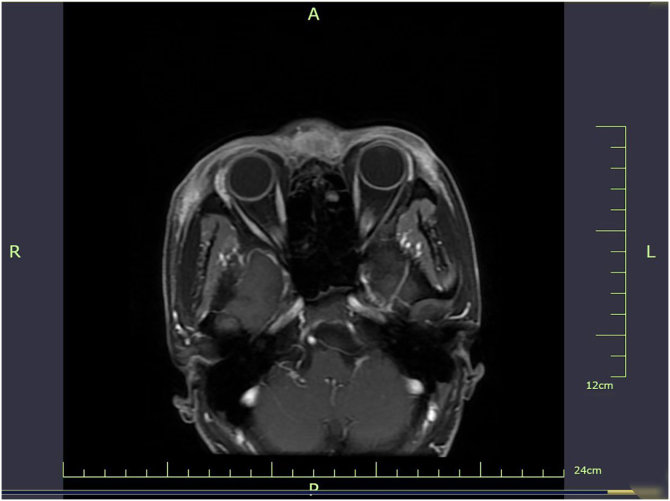
Enhanced MRI (pretreatment): The subcutaneous soft tissues of the frontotemporal region and periorbital area demonstrate thickening, with a maximum thickness of approximately 1.5 cm. Patchy foci of isointense T1 and slightly hyperintense T2 signals are identified, showing ill-defined margins and heterogeneous signal intensity. Post-contrast imaging reveals homogeneous enhancement of these lesions; on T2WI sequences, interspersed areas of hyperintense T2 signals are noted within the lesions, which also exhibit homogeneous enhancement following contrast administration.

**Figure 3:**
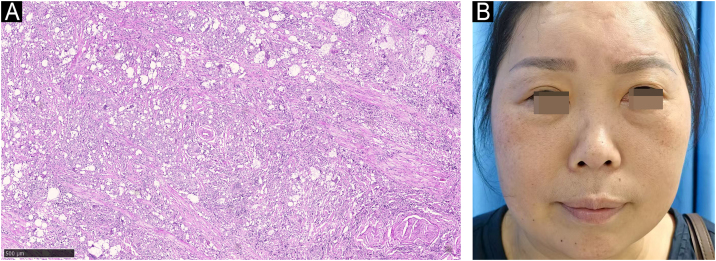
(A) Pathological picture, Hematoxilyn & eosin 4× magnification (pretreatment): Area of suspected cystic foreign body deposition, surrounded by a large number of epithelioid histiocytes and multinucleated giant cells. Some granulomas show nodular and diffuse distribution. Diffuse inflammatory cell infiltration (predominantly lymphocytes and plasma cells) is seen around the granulomatous nodules and in the stroma. (B) 3-weeks after treatment.

**Figure 4:**
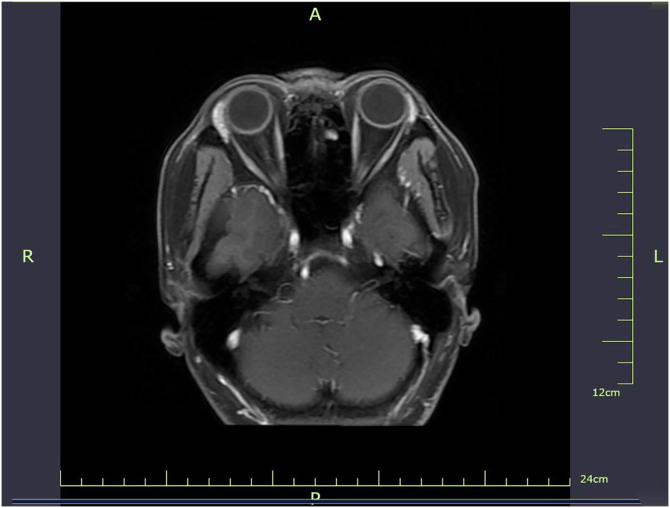
Enhanced MRI (3-weeks after treatment): The frontal soft tissues show slight swelling with heterogeneous slightly prolonged T1 and slightly prolonged T2 signals and ill-defined margins, with moderate enhancement noted on post-contrast imaging. Focal small patchy areas of slightly hyperintense DWI signals are seen in the local soft tissues. The frontal bone demonstrates linear patchy hyperintense DWI signals with insignificant changes in T2 and T1 signals; post-contrast enhancement is not well-defined, with suspected mild enhancement.

## Discussion

The incidence of FBG was reported to be 0.02%‒2.8% in 2017,^1^ however, the increasing demand for rejuvenation of facial fillers in the past 8-years drives this number a disappreciation of the real incidence. Intralesional corticosteroids, 5-Fluorouracil or excision have demonstrated benefits in patients with solitary granulomatous nodules. Systemic therapy, including oral prednisone, minocycline, allopurinol, colchicines, and cyclosporine, has been reported, but the side effects vary.^2^ There are scarce treatment options for patients with large-area edema.

Recently, applying JAK inhibitors to treat patients with granulomatous conditions has been reported and the improvement is remarkable.^3^, ^4^ FBG formation is a highly harmonious immunological response in order to isolate inert foreign materials that cannot be eliminated by immune cells. The inflammatory stimuli exhibit a Th1-driven immunological response^5^ and persist primarily through macrophage activation and accumulation.^6^ Although the immune pathomechanism of granulomatous inflammation is unclear, the signal pathways of the inflammatory disorders are common. Cytokines, such as Interferon γ (IFN-γ), IL (Interleukin)-2, IL-6, IL-12, IL17, IL-18, IL-23, Tumor Necrosis Factor-α (TNF-α), and T-cell chemokines, that are produced by both macrophages and lymphocytes, play a role in enhancing granulomatous inflammation. More than half of the cytokines mentioned above are confirmed to be dependent on the JAK signal transducer and activator of transcription pathway, which demonstrates JAK inhibitors can block the activity of the cytokines efficiently.^6^

Although not licensed for this use, upadacitinib, a JAK1 preferential inhibitor, seems to be promising for such severe swelling of GFB in our case, regarding its favorable safety and its rapid efficacy. To date, there is no standardized approach for FBG. Our observation provides a new perspective for the treatment of this challenging issue in the hope of adding to the finite strategy of therapy. Nevertheless, further controlled clinical studies with long-term follow-up is required to investigate the safety and efficacy of topical JAK inhibitors in GFB.

## ORCID ID

Mengying Duan: 0009-0007-5145-0545

Yi Wu: 0000-0002-6969-4552

Hong Shen: 0000-0003-4946-5848

## Data availability statement

The data that support the findings of this study are available from the corresponding author upon reasonable request.

## Ethical approval

The study was approved by the Institutional Ethics Committee of the Hangzhou Third People's Hospital (2025KA275).

## Ethics statement

The patient in this manuscript has given written informed consent to the publication of her case details.

## Research data availability

Does not apply.

## Financial support

None declared.

## Authors' contributions

Mengying Duan: Writing of the manuscript; Data collection; Analysis and interpretation.

Yi Wu: Data collection.

Hong Shen: Participation in the therapeutic conduct of the studied case.

Yeqin Dai: The study concept and design; Participation in the therapeutic conduct of the studied case; Final approval of the final version of the manuscript.

## Conflicts of interest

None declared.
